# Incidence of Corrective Procedures After Nonoperatively Managed Distal Radius Fractures in the Elderly

**DOI:** 10.5435/JAAOSGlobal-D-19-00159

**Published:** 2019-11-27

**Authors:** Nicholas P. Satariano, Gopal R. Lalchandani, Sarah E. Menchaca, Igor Immerman

**Affiliations:** From the Department of Orthopaedic Surgery (Dr. Satariano, Dr. Lalchandani, and Dr. Immerman), School of Medicine, University of California, San Francisco, and the UCSF School of Medicine (Ms. Menchaca), San Francisco, California.

## Abstract

Although the majority distal radius fractures in the elderly are initially managed nonoperatively, the true incidence of subsequent corrective surgery is unknown. The purpose of this study was to determine the incidence and predictors of corrective surgery after conservative management.

**Methods:**

*ICD-9* and Current Procedural Terminology codes were queried from the Medicare 5% sample to select patients aged 65 years and older undergoing nonsurgical treatment of distal radius fractures with a minimum 5-year follow-up. Rates of subsequent ipsilateral wrist surgery were correlated against patient age, sex, geographic region, and initial closed reduction.

**Results:**

Five thousand eighty patients with a mean age of 78.3 years were included. Fifty-five patients (1.1%) had undergone subsequent wrist surgery at a median time of 182 days after injury. The youngest cohort (65 to 69 years) had a significantly higher operation rate (1.9%, *P* = 0.007) than the oldest cohort (80+ years) (0.5%, *P* = 0.004). There was no notable difference in corrective procedures between sex, geographic region, and initial closed reduction.

**Discussion:**

Once surgical intervention is deemed unnecessary per standard guidelines, the data support successful nonsurgical management in a large majority of patients but highlight a small subset of younger patients who remain at increased risk of requiring additional surgery.

The societal and healthcare burden of distal radius fractures in the elderly is large and continues to increase. According to the US Census Bureau, the proportion of the public aged 65 years and older has continued to grow and is projected to account for over 20% of the cohort by 2050.^1^ Furthermore, analysis of the 2008 Nationwide Emergency Department Sample demonstrated a strong peak in the incidence of forearm and distal radius fractures in the elderly, with falls being a chief mechanism of injury.^2^ The overall incidence of distal radius fractures in patients older than 65 years is estimated at 372,000 cases per year and is expected to increase.^3^ In addition, current trends in the United States indicate an increasing use of internal fixation in the treatment of these injuries, particularly among hand surgeons.^4^ All these factors add up to increasingly higher costs to the healthcare system.

Recent literature has suggested that nonsurgical treatment of distal radius fractures in the elderly yields functional results equivalent to those of surgical treatment and with fewer complications.^3,5–7^ However, the rates of radiographic malunion are still high with nonsurgical treatment, and malunion is one of the most common complications seen after DR fractures.^3,8,9^ Despite the reported incidence of malunion, studies have shown that radiographic malunion is not predictive of lower function in highly active seniors or super-elderly patients (≥80) and in fact have documented an extremely low rate of surgery (<1%) after initially nonsurgical treatment.^10,11^ This is in contrast to the findings of Brogren et al who reported an increased rate of disability associated with distal radius malunion using an age-adjusted prospective cohort.^12,13^ Kilic et al^14^ found a 10% rate of corrective osteotomies in elderly patients treated nonoperatively, although the indications for surgery were not specified. In addition to these seemingly conflicting outcomes, much of the previous literature was conducted on relatively small study cohorts and over short periods. Furthermore, there is a paucity of data on the demographic and patient-related factors of those requiring secondary surgery.

The purpose of this study was to determine the incidence of corrective surgery after nonoperatively managed distal radius fractures in the elderly using a large nationwide claims database with a follow-up period of at least 5 years. We hypothesized that a small minority of younger patients with distal radius fractures would be at higher risk of undergoing secondary surgery.

## Methods

This study used administrative claims data from the Medicare 5% sample claims Standard Analytic Files database (PearlDiver Technologies Inc, Warsaw, IN; www.pearldiverinc.com) composed of a representative sample of all patients with Medicare records. At the time of query (June 2017), information was available from 2007 through 2014. The database was searched using *ICD-9* (*International Statistical Classification of Diseases-9*) diagnosis codes for distal radius fracture and Current Procedural Terminology (CPT) codes for nonsurgical management of distal radius fractures to identify patients who sustained a distal radius fracture and were subsequently managed nonoperatively from 2007 to 2014 (Table 1). Nonsurgical treatment was defined either by a CPT code signifying closed treatment of a distal radius fracture or by a combination of appropriate *ICD-9* code and a code for the application of a splint or a cast (Table 1). Patients were included if they were aged 65 years and older and had a minimum of 5-year follow-up data from the initial injury with laterality specified in initial codes. Exclusion criteria included receiving surgical management as defined by CPT codes (Table 1) within 6 weeks of injury.

**Table 1:** *ICD-9* and CPT Codes for Inclusion and Exclusion Criteria

	Definition
*ICD-9* DRF	
813.4	Fracture, lower end of forearm, closed; unspecified
813.41	Colles' fracture, closed
813.42	Other fracture of distal end of radius (alone), closed
813.44	Fracture, radius with ulna, lower end, closed
813.45	Torus fracture of radius (alone), closed
CPT inclusion codes	
29065	Application, cast; figure-of-8, shoulder to hand (long arm)
29075	Application, cast; figure-of-8, elbow to finger (short arm)
29085	Application, cast; figure-of-8, hand and lower forearm (gauntlet)
29105	Application of long arm splint (shoulder to hand)
29125	Application of short arm splint (forearm to hand); static
29126	Application of short arm splint (forearm to hand); dynamic
25600	Closed treatment of DRF, without manipulation
25605	Closed treatment of DRF, with manipulation
CPT exclusion codes	
25606	Percutaneous skeletal fixation of distal radial fracture or epiphyseal separation
25607, 25608, 25609	Open treatment of distal radial extra-articular fracture or epiphyseal separation, with internal fixation
20690, 20692, 20693	External fixation

CPT = Current Procedural Terminology, DRF = distal radius fracture

Demographic data recorded included age, sex, and region of the country (Table 2). Closed manipulation at time of initial injury was also recorded using the respective CPT for closed treatment with (25605) or without (25600) manipulation (Table 2). A subsequent wrist surgery was defined as a procedure occurring at least 6 weeks from the initial injury and identified by a variety of CPT codes (Table 3) with laterality matching the initial side of injury. Owing to the nature of the data set, procedures that occurred in 10 or fewer patients were not specified to maintain patient anonymity. The average time from injury to corrective procedure and the overall incidence of corrective surgery was calculated at 5 years of follow-up. The insurance reimbursement associated with each procedure encompassing the hospital encounter including anesthesia, radiology, and facility fees was also tabulated.

**Table 2:** Demographics and Closed Manipulation Data

Factor	Total	Percent
Age		
65-69	986	19.4%
70-74	1076	21.2%
75-79	1186	23.3%
80+	1930	38.0%
Region		
Midwest	1596	31.4%
Northeast	1440	28.3%
South	1558	30.7%
West	497	9.8%
Sex		
Female	4450	87.6%
Male	630	12.4%
Closed manipulation		
Done	2436	48.0%
Not done	2667	52.5%

**Table 3:** CPT Codes for Secondary Procedure

CPT Secondary Procedure Codes	Definition
25606	Percutaneous skeletal fixation of distal radial fracture or epiphyseal separation
25607, 25608, 25609	Open treatment of distal radial extra-articular fracture or epiphyseal separation, with internal fixation
25400, 25405, 25415, 25420, 25425, 25426	Repair of distal radius/ulna malunion/nonunion
25515, 25525, 25526	ORIF radial shaft
25545	ORIF ulnar shaft
25350, 25355, 25360, 25365, 25370, 25375	Radial and/or ulnar osteotomy
25390, 25391, 25392, 25393	Radius and/or ulnar osteoplasty
25240	Excision distal ulna partial/complete (Darrach type)
25230	Radial styloidectomy
25651, 25652	Ulnar styloid fixation
25337, 25830	DRUJ procedures
25800, 25805, 25810, 25820, 25825	Wrist arthrodesis
25332, 25441, 25442, 25446, 25449	Wrist arthroplasty
29840, 29843, 29844, 29845, 29846	Wrist arthroscopy
20690, 20692, 20693	Application of external fixator
25999	Unlisted procedure (when associated with *ICD-9*)

CPT = Current Procedural Terminology, DRUJ = distal radial ulnar joint, ORIF = open reduction and internal fixation

For statistical analysis, the variables of age, sex, region of the United States, and closed manipulation were tested against the presence of a subsequent wrist surgery using univariate and multivariate analysis. Kaplan-Meier methods were used to describe the survival of conservatively managed patients without a secondary procedure over time. Statistical analyses were done using Microsoft Excel (Microsoft Corp, Redmond, WA).

## Results

Our query of the PearlDiver database for distal radius fractures managed nonoperatively yielded 5080 patients with an average age of 78.3 years with 5-year follow-up and complete documentation of all demographic data. Demographically, there was an anticipated overwhelming female majority and roughly equal numbers by region. Nearly half of patients underwent a closed manipulation at time of initial injury (Table 2).

**Figure 1:**
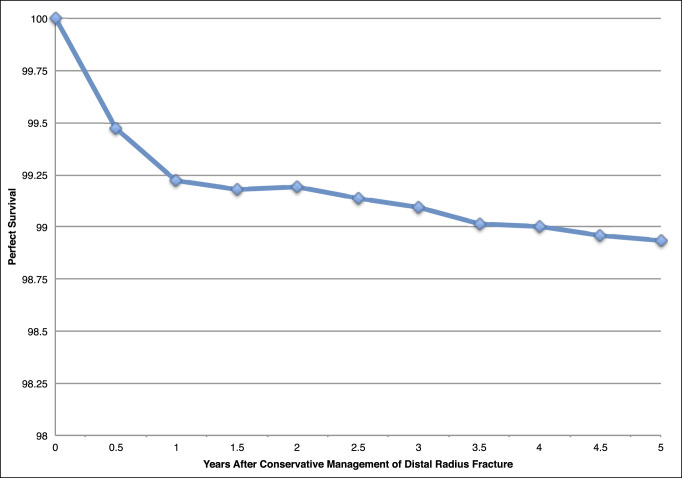
Identification: Kaplan-Meier survival of conservatively managed patients without secondary procedure.

After 5 years, there was an overall 1.1% incidence of subsequent wrist surgery occurring at an average time of 182 days from initial injury. The survival of conservatively managed patients without requiring a secondary procedure is shown in Figure 1. On stratification by age, region, sex, and closed manipulation, only age was found to be a significant risk factor for need of corrective procedure in both univariate and multivariate analysis. In the youngest cohort aged 65 to 69 years, there was an observed 1.9% incidence of secondary surgery. Each ensuing age group displayed a decreasing frequency of subsequent procedure with the lowest incidence reported in the oldest cohort aged 80 + years at only 0.5% (Tables 4 and 5).

**Table 4:** Incidence of Corrective Procedures by Demographics and Closed Manipulation—Univariate Analysis

Factor	# Procedure	% Procedure	Odds Ratio	*P*
Age				
65-69	19	1.9%	2.27	**0.007**
70-74	15	1.4%	1.44	0.197
75-79	12	1.0%	0.94	0.392
80+	9	0.5%	0.33	**0.004**
Region				
Midwest	15	0.9%	0.82	0.322
Northeast	15	1.0%	0.95	0.393
South	17	1.1%	1.01	0.398
West	8	1.6%	1.58	0.196
Sex				
Female	46	1.0%	0.72	0.268
Male	9	1.4%	1.39	0.268
Closed manipulation				
Done	32	1.3%	1.57	0.101
Not done	23	0.9%	0.64	0.101

**Table 5:** Incidence of Corrective Procedures by Demographics and Closed Manipulation—Multivariate Analysis

Factor or Variable	Odds Ratio	Confidence Interval	*P*
Age			
65-69	1.00	Reference	N/A
70-74	0.64	0.35-1.16	0.147
75-79	0.68	0.37-1.17	0.164
80+	0.39	0.19-0.76	**0.008**
Region			
Midwest	1.00	Reference	N/A
Northeast	1.10	0.59-2.04	0.770
South	1.41	0.80-2.51	0.236
West	1.83	0.87-3.70	0.099
Sex			
Female	1.00	Reference	N/A
Male	1.01	0.50-1.84	0.985
Closed manipulation			
Done	1.00	Reference	N/A
Not done	0.75	0.48-1.17	0.205

Of the surgeries done, nonunion/malunion repair was most common at 29.1%, followed by radial osteotomy (21.8%), and distal ulnar resection/Darrach type procedure (20.0%) (Table 6). There was no notable association noted between type of procedure and demographic or closed manipulation data. Regarding economic burden to the healthcare system, the average insurance reimbursement of an additional procedure was $4636.

**Table 6:** Type of Corrective Procedures Done

Procedure	Total	Percent
Nonunion or malunion repair	16	29.1%
Osteotomy of radius	12	21.8%
Distal ulnar resection/Darrach type	11	20.0%
DRUJ reconstruction	≤10	≤10.0%
Wrist arthrodesis	≤10	≤10.0%
Wrist arthroplasty	≤10	≤10.0%
Wrist arthroscopy	≤10	≤10.0%

DRUJ = distal radial ulnar joint

## Discussion

We used a large nationwide administrative claims database to identify and retrospectively follow a cohort of Medicare patients who were managed nonoperatively for their distal radius fractures. Similar to how other studies have used a Medicare 5% sample claims database to longitudinally track patients after total joint arthroplasty,^15–17^ we aimed to identify the true incidence of corrective surgery after nonoperatively managed distal radius fractures and to investigate risk factors for this specific subgroup.

Our study demonstrated that among elderly patients aged 65 years and older who undergo nonsurgical management for a distal radius fracture, there is a 1.1% incidence at 5-year follow-up for subsequent wrist surgery. This finding is similar to previous outcomes in smaller cohort s by Clement et al and Nelson et al who reported corrective procedure incidence at less than 1%.^10,11^ The fact that the overwhelming majority of our patients did not require a subsequent surgery also reinforces the body of literature supporting conservative management in the elderly as having low reoperation rates.^3,5–7^

With respect to demographic data, we did find age as a notable predictor of need for future corrective surgery. Patients in the youngest category aged 65 to 69 years had a statistically significant increased risk of undergoing a subsequent procedure compared with every older cohort thus validating our hypothesis. Our finding is in line with the data from Brogden et al who showed younger age as being associated with a worse functional score at 1 year after injury and Clement et al who reported the effect of malunion as diminishing with increasing age.^10,13^ This difference may be explained by the variations in activity level depending on patient age. Younger patients may be more active than their older counterparts and thus tolerate less functional limitations related to any malalignment that may result from closed treatment of a distal radius fracture. Moreover, surgeons may be more likely to offer elective surgery to healthier younger patients.

In regard to closed reduction at time of injury, patients who underwent closed manipulation had a slightly higher rate of undergoing subsequent surgery than patients treated without manipulation, but statistical significance was not reached. Although it is likely that patients requiring manipulation may be presenting with higher initial displacement and thus are more likely to require surgery, overall, our data did not show a statistical difference. Although radiographic parameters were not evaluated in this study, previous work has shown that radiographic malunion is not a good predictor of outcome in the elderly.^10,11,18^ This finding may help explain why need for a reduction based on initial displacement did not markedly increase the risk of later operation.

With respect to the types of corrective procedures done, we found that CPT codes for nonunion/malunion repair were most common followed by radial osteotomy and distal ulnar resection. Surgical correction of a malunion may be represented by a variety of CPT codes including either osteotomy or malunion repair, and these represent the bulk of corrective procedures in this cohort. Ulnar shortening, which may be easier to do but can only correct the ulnar positivity associated with DR malunion, was done in only 20% of cases. There were several other surgeries reported including distal radioulnar joint reconstruction, arthrodesis, arthroplasty, and arthroscopy; however, these occurred much more infrequently, and because of the nature of the database, query could only be reported as less than 10 occurrences. Our observation that patients most often require a malunion repair supports previous literature highlighting the prevalence of malunion with conservative management of distal radius fractures.^3,8,9^

An additional finding unique to this study was the reported average time to surgery from initial injury. An overwhelming majority of secondary procedures took place in the 1st year, on average occurring nearly 6 months after injury as demonstrated by the Kaplan-Meier survival curve (Figure 1). From a prognostic standpoint, it appears that if patients can pass 1 year after injury with no notable functional limitations and sufficient signs of radiographic healing, they can be encouraged that they will most likely not require an additional intervention.

Although one of the advantages of this study is the vast patient cohort pulled from all regions of the country, there are limitations to relying on a large administrative database. The analysis hinges heavily on the accuracy of procedural and diagnostic coding. Although miscoding has been reported as not an uncommon occurrence, in theory, this should only represent a minority of our cohort.^19^

The largest limitation of our study is inability to assess the radiographic severity of the injury and a lack of a surgical comparison group. Therefore, we are unable to assess what percentage of our cohort had surgical indications or radiographic malunion. Based on the literature, we can presume that as many as 17% to 23.5% of these fractures healed with radiographic malunion.^8,20,21^ In addition, the fact that nearly half of the fractures were managed with manipulation supports our assumptions that many of these patients presented with displaced fractures.

In the literature, treatment recommendations such as the American Academy of Orthopaedic Surgeons clinical guidelines and appropriate use criteria exist that can help guide management.^22^ We have to presume that the physicians managing our patients followed the guidelines to at least some extent, and therefore, few of the patients in the nonsurgical cohort are likely to have markedly displaced or severe intra-articular injuries. In addition, the use of surgery within 6 weeks of initial injury was selected as exclusion to criteria to ensure we captured only those injuries that truly merited nonsurgical management.

Despite these limitations, this study's main strength is the utilization of a larger, more comprehensive patient cohort than previous work to accurately report the true incidence of nonoperatively managed distal radius fractures in the elderly patients who go on to require a subsequent surgery. In addition, the ability to track patients for 5 years after injury represents one of the longest follow-up periods in the literature to date. To further understand the natural history of distal radius fractures in the elderly, future studies could explore the specific indications for subsequent procedures, evaluating pertinent radiographics and patient function.

The goal of this study was to determine the true proportion of elderly patients who require corrective wrist surgery after the initial nonsurgical treatment of a distal radius fracture. As anticipated, there was only a small percentage (1.1% at 5 years) that underwent a subsequent wrist surgery. This specific cohort included markedly more young patients (aged 65 to 69 years) with an incidence of 1.9% at 5-year follow-up. In addition, most patients who required a corrective procedure did so within 1 year of injury. Overall, the findings demonstrate that once nonsurgical management of a distal radius fracture in an elderly patient is chosen, it is likely to be successful with a low reoperation rate.
